# 1943. RSV Prophylaxis for 29-34 Gestational Preemies. Are Current Guidelines Justified? Lessons from an Israeli Multicenter Off-seasonal Study

**DOI:** 10.1093/ofid/ofad500.097

**Published:** 2023-11-27

**Authors:** Keren Armoni Domany, Avigdor Mandelberg, Nitzan Burrack, Inbal Tripto Golan, Kamal Masarweh, Mika Rochman, Dario Prais, Meirav Mor, Moran Weinberger Opek, Elias Nasrallah, Orli Megged, Rachel Shatzman Steuerman, Michal Stein, Zohar Steinberg, Shereen Shehadeh, Diana Tasher

**Affiliations:** wolfson medical center, holon, HaMerkaz, Israel; Department of Pediatrics, Edith Wolfson Medical Center, Holon /Sackler School of Medicine, Tel-Aviv University, Tel-Aviv, Holon, HaMerkaz, Israel; Pediatric Pulmonary Unit, Soroka University Medical Center, Beer-Sheva, Israel, BEER SHEVA, HaDarom, Israel; Pediatric Pulmonary Unit, Soroka University Medical Center, Beer-Sheva, Israel, BEER SHEVA, HaDarom, Israel; Pediatric Pulmonary Institute and CF Center, The Ruth Rappaport Children's Hospital, Haifa, Israel, HAIFA, HaZafon, Israel; Pediatric Pulmonology Unit, Tel-Aviv Sourasky Medical Center, Israel, TEL AVIV, Tel Aviv, Israel; Pulmonary Institute, Schneider Children’s Medical Center, Israel, PETAH TIKVA, HaMerkaz, Israel; Schneider Children's Medical Center of Israel; Tel Aviv University, Petach Tikva, Israel; Department of Pediatrics, Samson Assuta Ashdod University Hospital, Ashdod, Israel 12. Saint Vincent De Paul Hospital, Israel, Ashdod, HaDarom, Israel; Saint Vincent De Paul Hospital, Israel, Nazeret, HaZafon, Israel; Shaare Zedek Medical Center (affiliated with Hebrew University, Hadassah School of Medicine), Jerusalem, Israel., Jerusalem, Yerushalayim, Israel; Pediatric Infectious Disease Unit, Sheba Medical Center, Israel, Kfar Saba, HaMerkaz, Israel; Hillel Yaffe Medical Center, Hadera, HaMerkaz, Israel; Department of Pediatrics, Carmel Medical Center, Haifa, Israel., Afula, HaZafon, Israel; Department of Pediatrics, Carmel Medical Center, Haifa, Israel., Afula, HaZafon, Israel; Pediatric Infectious Diseases Unit, Edith Wolfson Medical Center, Holon /Sackler School of Medicine, Tel-Aviv University, Tel-Aviv, Holon, HaMerkaz, Israel

## Abstract

**Background:**

Respiratory syncytial virus (RSV) prophylaxis provided during the winter to infants born at 29–34-week gestational age (wGA) is controversial. We utilized the unprecedented opportunity of high load off-season RSV admissions with no prophylaxis given during the summer of 2021, to assess the impact of withholding palivizumab on the relative burden of hospitalized 29-34 wGA infants.

**Methods:**

This was a national multi-center observational retrospective study in 11 medical centers in Israel. We included infants under 1 year-old, who were hospitalized with RSV between November 2017 and August 2021. Demographics and clinical data were extracted from the medical records. We categorized our cohort into a seasonal admissions group (November- March) when palivizumab was indicated, and an off-season admissions group (April-October) when palivizumab was not indicated. The primary outcome was the proportion of 29-34 wGA hospitalizations in each group. Secondary outcomes included clinical severity parameters.

**Results:**

We included 4,340 children in our study; 57% were males, 61% were Jewish, and the median age at admission was 2.9 months (IQR 1.4-6.4). A total of 3,296 infants were admitted during the RSV season, and 1,044 were admitted off-season. The proportion of 29-34 wGA preemies was significantly higher in the off-season admissions group compared to the in season patients (7% vs 2.1%, p< 0.001). We found a significantly higher prevalence of Jewish (77% vs 57%, p=0.001) and higher socioeconomic status (55% vs 45%, p< 0.001) children in the group admitted off-season compared to those in season. Logistic regression analysis revealed that the relative-odds of 29-34 wGA preemies to be hospitalized off-season was 2.67 (95% CI: 1.8-3.9, p< 0.001) fold higher than in season, independent of ethnicity, socioeconomic-status, gender, age, or institution. Clinical severity parameters did not differ significantly between the groups.

**Figure:**
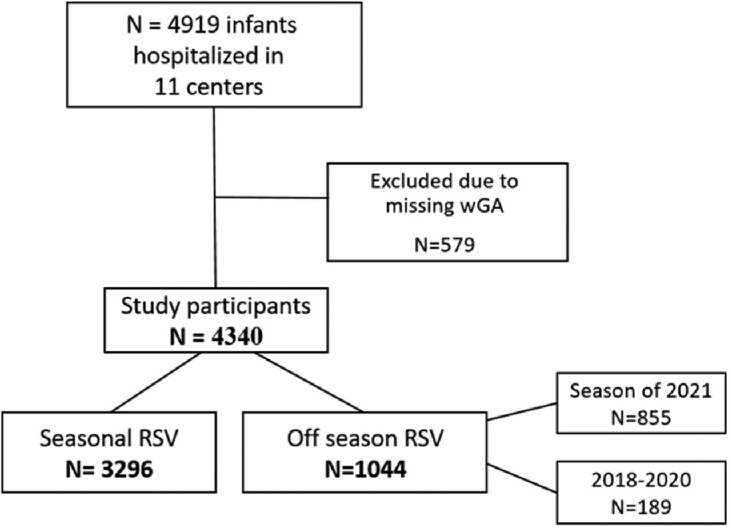

Flow chart of study participants

**Figure:**
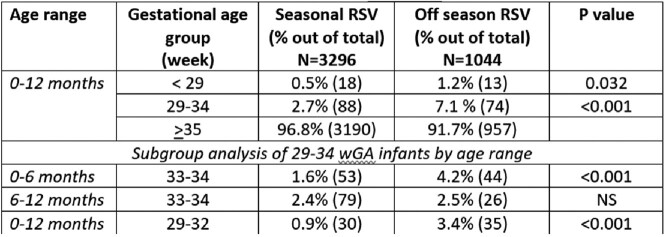

The proportion of 29-34 wGA preterm infants out of total admissions categorized into seasonal and out of season RSV infections

**Figure:**
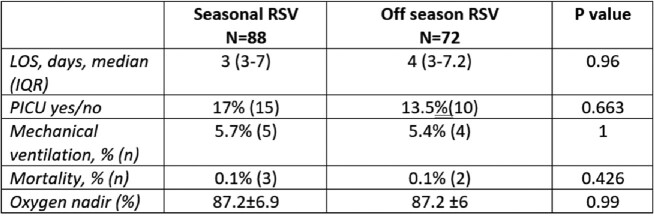

Clinical severity in the subgroup of 29-34 wGA preterm infants

**Conclusion:**

Our results reveal a significant higher proportion of 29-34 wGA preemies among the children hospitalized during off-season periods, when RSV immunoprophylaxis was not indicated. These findings may support the current broad Israeli policy of administration.

**Disclosures:**

**All Authors**: No reported disclosures

